# Predictors of Low Voltage Zone and Sex Differences in Low Voltage Zone Distribution in Patients with Atrial Fibrillation

**DOI:** 10.31083/j.rcm2411324

**Published:** 2023-11-23

**Authors:** Yu Xin, Fei Hang, Yongquan Wu

**Affiliations:** ^1^Department of Cardiovascular Medicine, Beijing Anzhen Hospital, Capital Medical University, 100029 Beijing, China

**Keywords:** atrial fibrillation, high-density electro-anatomic mapping, left atrial low voltage zone, sex differences

## Abstract

**Background:**

Previous studies have revealed the left atrial (LA) low voltage zone (LVZ) are tightly linked to the recurrence of atrial fibrillation (AF). Furthermore ablation that targets the LA LVZ can improve patient prognosis. The aim of this study was to identify potential clinical predictors of the LA LVZ, to investigate possible sex differences in the distribution of LA LVZ, and to examine the relationship between LA LVZ and AF recurrence.

**Methods:**

A total of 108 patients who underwent AF catheter ablation and LA high-density electro-anatomic mapping were enrolled in the study. Of these, 56 patients with LA LVZ ≥5% were assigned to the LVZ group, while the remaining 52 patients with LA LVZ <5% were assigned to the non-LVZ group. Clinical characteristics and laboratory results for all patients were collected and compared between the two groups.

**Results:**

Multivariate logistic regression analysis revealed that persistent AF (odds ratio [OR] = 4.563, 95% confidence interval [CI]: 1.194–17.431, *p* = 0.026), left atrial volume (LAV, OR = 1.030, 95% CI: 1.001–1.061, *p *= 0.044) and brain natriuretic peptide (BNP, OR = 1.010, 95% CI: 1.002–1.019, *p *= 0.015) were independent predictors for the presence of LA LVZ. In addition, female sex (OR = 7.161, 95% CI: 1.518–33.792, *p* = 0.013), LAV (OR = 1.028, 95% CI: 1.002–1.055, *p* = 0.035) and BNP (OR = 1.009, 95% CI: 1.001–1.016, *p *= 0.018) were independent predictors of severe LA LVZ (LA LVZ >20%). The extent of LVZ was significantly greater in females than in males (32.8% ± 15.5% *vs*. 23.5% ± 12.7%, *p *= 0.021), especially in the anterior (34.5% ± 16.7% *vs*. 20.0% ± 16.4%, *p *= 0.003) and septal (44.9% ± 17.1% *vs*. 29.0% ± 18.9%, *p *= 0.004) walls. During follow-up, AF recurrence was significantly higher in patients with LA LVZ than in those without LA LVZ (31.3% *vs*. 12.8%, respectively, *p *= 0.023).

**Conclusions:**

In this study cohort, persistent AF, LAV and BNP were independent predictors of LA LVZ. Furthermore, female sex, LAV and BNP were independent predictors of severe LA LVZ. Females had a significantly greater extent of LVZ than males, especially in the anterior and septal walls. Patients with LA LVZ had a higher risk of recurrent AF.

## 1. Introduction

Atrial fibrillation (AF) is one of the most common cardiac arrhythmias worldwide [1, 2]. The risk of stroke, heart failure, and even cardiac death in patients with AF is significantly increased, thus severely impacting their quality of life and causing financial burden [3, 4].

The mechanism of AF is still controversial and has yet to be fully elucidated. It has been proposed that left atrial (LA) remodeling is strongly associated with the progression of AF, and that LA fibrosis is a major factor leading to LA remodeling [5, 6]. Many studies have shown that the LA low-voltage zone (LVZ), which can be identified by high-density electro-anatomic mapping, can be used to evaluate LA fibrosis [7]. Earlier work confirmed that the presence of LA LVZ has a negative impact on the success of AF catheter ablation and increases AF recurrence during follow-up [8, 9]. Targeting the LA LVZ has been shown to suppress the recurrence of AF [10, 11]. Identification of predictive factors for the presence of LA LVZ before the ablation procedure can therefore help to select the appropriate strategy and to evaluate patient prognosis.

The incidence, clinical manifestations and prognosis of AF differs by sex. Although the incidence of AF is lower in females, women are more likely to experience symptoms and AF recurrence [12, 13, 14]. The etiology underlying sex differences in AF have not been fully elucidated. A histological study found that females experience significantly more aggravation of fibrosis remodeling than males [13]. Exploration of the distribution of LA LVZ according to sex therefore seems warranted.

The aim of the present study was to investigate potential clinical predictors of LA LVZ, as well as sex differences in the distribution of LA LVZ. The relationship between LA LVZ and AF recurrence was also further evaluated in this study.

## 2. Methods

### 2.1 Study Population

We enrolled 108 patients with paroxysmal or persistent AF who underwent AF catheter ablation and LA high-density electro-anatomic mapping at the Beijing Anzhen Hospital from January 2021 to January 2023. The exclusion criteria were: (1) age <18 years, (2) history of catheter ablation, cryo-balloon ablation, or other LA surgeries, (3) severe structural heart disease, (4) acute or chronic inflammatory diseases. All patients provided signed informed consent.

### 2.2 Data Collection

Baseline clinical characteristics for all patients were collected, including age, sex, body mass index, type of AF, comorbidities, history of smoking and drinking and other indicators. Laboratory results collected for the study included red blood cell count, white blood cell count, platelet count, as well as the levels of alanine aminotransferase, aspartate transaminase, brain natriuretic peptide (BNP), total bilirubin (TBIL), direct bilirubin, estimated glomerular filtration rate (eGFR), uric acid (UA), high-density lipoprotein cholesterol, low-density lipoprotein cholesterol, complement component 1q, homocysteine and high-sensitivity C-reactive protein. Echocardiographic parameters including left ventricular ejection fraction (LVEF), left atrial diameter (LAD), and left ventricular end-diastolic dimension were also collected. The PR interval and corrected QT interval (QTc) of preoperative electrocardiograms were also measured.

### 2.3 High-Density Electro-Anatomic Mapping and Radiofrequency Catheter Ablation 

All patients underwent transesophageal echocardiography to rule out LA thrombus prior to catheter ablation. Bilateral femoral vein punctures were performed under local anesthesia. A 6-F introducer sheath (Medtronic, Minneapolis, MN, USA) and a 7-F introducer sheath were then inserted into the left femoral vein. The right femoral vein was inserted with two 8.5-F long SL1 sheaths (St. Jude Medical, St. Paul, MN, USA) for transseptal puncture. A 10-polar diagnostic catheter was delivered into the coronary sinus via the 6-F introducer sheath, followed by two transseptal punctures. A High-Density Mapping Catheter (Biosense Webster, Diamond Bar, CA, USA) and an STSF ablation catheter (Biosense Webster, Diamond Bar, CA, USA) were delivered into the LA via the 8.5-F long SL1 sheaths. Mapping was performed using PentaRay under the guidance of a three-dimensional electro-anatomical mapping system (Biosense Webster, Inc., Diamond Bar, CA, USA), with the bipolar voltage recorded automatically at each point. After the completion of mapping, all patients underwent circumferential pulmonary vein isolation (CPVI) in a power control mode of 45 W and following standard protocols. If AF persisted after CPVI, additional atrial ablation methods (cavotricuspid isthmus ablation, LA linear ablation, or LVZ ablation) based on the high-density voltage mapping results were performed in order to terminate AF episodes. During this procedure, heparin was administered continuously to maintain a target activated clotting time of between 300–350 s.

**Fig. 1.**
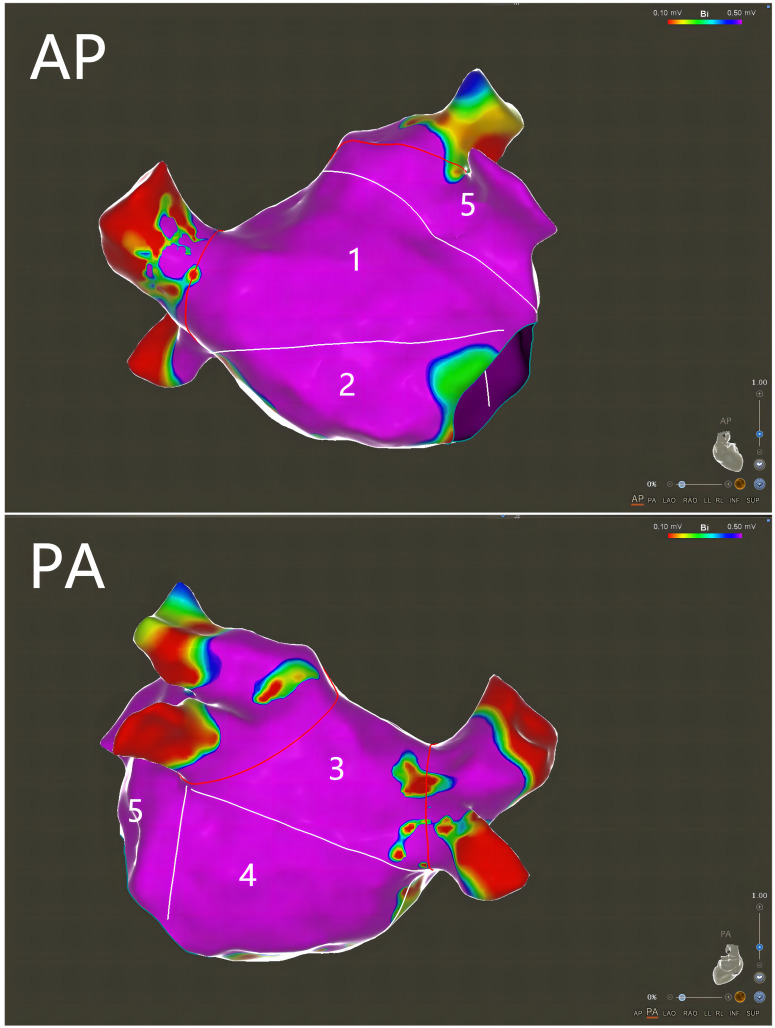
**Left atrial (LA) low voltage zone (LVZ) assessment by high-density voltage mapping**. Anterior-posterior (AP) and posterior-anterior (PA) views show how the LA was divided for analysis of the LA LVZ distribution (1 = anterior wall, 2 = septal wall, 3 = posterior wall, 4 = inferior wall, 5 = lateral wall).

The analysis of each electrogram was performed offline. Data acquisition was limited by internal point filter software to ensure that only mapping points within a distance of 7 mm from the acquired LA shell were selected for voltage mapping. Low voltage was defined as a bipolar voltage <0.5 mV in sinus rhythm, and <0.3 mV in AF rhythm [15, 16]. The total LA surface area was defined as the entire area excluding the pulmonary vein and tricuspid valve. The global area of LA LVZ was measured using the Carto system and then expressed as a percentage of the total LA surface area. From the mapping results, patients with LA LVZ ≥5% were classified as LVZ and those with LA LVZ <5% as non-LVZ, respectively. Moreover, LA LVZ >20% was considered to be severe LA fibrosis [17]. The LA was divided into 5 regions (anterior, septal, posterior, inferior, and lateral walls) based on a previously described method [18]. The same method was used to measure the surface area and LVZ area of each region, with the volume of LA (LAV) measured at the same time. The mapping results are presented in Fig. 1.

### 2.4 Follow-Up

Regular follow-up visits were conducted at 3 months, 6 months, 12 months, or longer after radiofrequency catheter ablation (RFCA) in outpatient clinics, and patients with less than 6 months of follow-up were excluded. At each visit, patients were asked to give details of their symptoms. A 12-lead electrocardiogram (ECG) or 24 h holter monitoring was also performed. For this study cohort, AF recurrence was defined as the duration of an AF or atrial flutter >30 s captured on 12-lead ECG or 24 h holter monitoring after a 3-month blanking period.

### 2.5 Statistical Analysis

All statistical analyses were performed using SPSS software (Version 25, IBM Corp., Armonk, NY, USA). Continuous data with normal distribution were presented as the mean ± standard deviation and were analyzed using the independent sample *t*-test. Data that were not normally distributed were presented as the median with interquartile range and analyzed using the Mann-Whitney U-test. Categorical variables were described as frequencies or percentages and were compared between groups using the chi-squared test or Fisher’s exact test. Multivariate logistic regression analysis was used to identify possible predictors of LA LVZ. The multivariate model included variables with a *p* value of <0.05 in the univariate model, as well as variables with* p *value > 0.05 that were reported to have predictive value for the presence of LA LVZ in previous studies. AF recurrence-free survival in the LVZ and non-LVZ groups was analyzed using Kaplan-Meier curves and compared using the log-rank test. A Cox regression analysis model was used to investigate risk factors for AF recurrence. Spearman’s correlation was used to assess the associations between continuous variables. Two-sided *p* values <0.05 were considered statistically significant.

## 3. Results

### 3.1 Baseline Clinical and Laboratory Characteristics

A total of 108 participants with paroxysmal or persistent AF were enrolled in the study, including 56 patients in the LVZ group and 52 patients in the non-LVZ group. Of these, 75 (69.4%) were male. The baseline clinical and laboratory characteristics are shown in Tables 1,2. Patients in the LVZ group had a higher proportion of persistent AF (*p <* 0.0001) and heart failure (HF) (*p* = 0.028), as well as significantly higher levels of BNP (*p <* 0.0001), TBIL (*p* = 0.004), eGFR (*p* = 0.034) and UA (*p* = 0.045) compared to non-LVZ patients. Additionally, patients in the LVZ group had longer QTc (*p* = 0.012) than those in the non-LVZ group. Echocardiography showed that patients with LVZ had larger LAD (*p <* 0.0001) and LAV (*p <* 0.0001), but lower LVEF (*p* = 0.009) than non-LVZ patients.

**Table 1.** **Baseline clinical characteristics**.

Characteristic	Total (n = 108)	LVZ (n = 56)	Non-LVZ (n = 52)	*p* value
Age (years)	58.77 ± 9.86	60.48 ± 8.38	56.92 ± 11.02	0.061
Female, n (%)	33 (30.6)	18 (32.1)	15 (28.8)	0.710
BMI, kg/$m^{2}$	25.94 ± 2.76	25.96 ± 2.90	25.92 ± 2.62	0.949
Persistent AF, n (%)	43 (39.8)	37 (66.1)	6 (11.5)	<0.0001
HTN, n (%)	56 (51.9)	31 (55.4)	25 (48.1)	0.449
DM, n (%)	37 (34.3)	19 (33.9)	18 (34.6)	0.940
CAD, n (%)	13 (12.0)	9 (16.1)	4 (7.7)	0.181
HF, n (%)	10 (9.3)	9 (16.1)	1 (1.9)	0.028
Stroke, n (%)	13 (12.0)	8 (14.3)	5 (9.6)	0.456
Smoking, n (%)	20 (18.5)	13 (23.2)	7 (13.5)	0.192
Drinking, n (%)	17 (15.7)	12 (21.4)	5 (9.6)	0.092
LAD, mm	39.87 ± 5.04	41.89 ± 4.95	37.69 ± 4.18	<0.0001
LVEF, %	62.04 ± 6.93	60.38 ± 7.88	63.83 ± 5.25	0.009
LVEDD, mm	48.01 ± 4.94	48.66 ± 5.01	47.31 ± 4.81	0.156
LAV, mL	106.37 ± 28.63	119.62 ± 26.31	92.11 ± 23.93	<0.0001
PR interval, ms	162.45 ± 19.57	165.85 ± 19.74	158.78 ± 18.90	0.061
QTc, ms	438.40 ± 24.37	444.01 ± 22.30	432.35 ± 25.26	0.012

AF, atrial fibrillation; BMI, body mass index; CAD, coronary artery disease; DM, diabetes mellitus; HTN, hypertension; HF, heart failure; LAD, left atrial diameter; LAV, left atrial volume; LVZ, low voltage zone; LVEDD, left ventricular end-diastolic dimension; LVEF, left ventricular ejection fraction; QTc, corrected QT interval.

**Table 2.** **Baseline laboratory characteristics**.

Characteristic	Total (n = 108)	LVZ (n = 56)	Non-LVZ (n = 52)	*p* value
RBC, ${\mathrm{10}}^{12}$/L	4.78 ± 0.50	4.76 ± 0.51	4.80 ± 0.49	0.659
WBC, ${\mathrm{10}}^{9}$/L	6.48 ± 1.28	6.60 ± 1.38	6.34 ± 1.18	0.299
PLT, ${\mathrm{10}}^{9}$/L	209.44 ± 43.80	205.59 ± 46.05	213.58 ± 41.28	0.346
Hb, g/L	149.00 ± 15.57	149.48 ± 16.00	148.48 ± 15.23	0.740
MCV, fL	90.93 ± 3.71	91.41 ± 3.93	90.42 ± 3.43	0.167
MCH, pg	31.14 ± 1.40	31.33 ± 1.49	30.94 ± 1.29	0.146
MCHC, g/L	342.49 ± 8.35	342.75 ± 8.99	342.21 ± 7.69	0.739
HCT, %	43.47 ± 4.41	43.55 ± 4.57	43.38 ± 4.28	0.843
RDW-SD, fL	42.49 ± 2.35	42.70 ± 2.55	42.28 ± 2.12	0.352
RDW-CV, %	13.00 ± 0.59	12.97 ± 0.64	13.03 ± 0.53	0.578
MPV, fL	10.06 ± 1.16	9.98 ± 1.17	10.15 ± 1.17	0.419
PCT, %	0.21 ± 0.04	0.20 ± 0.04	0.22 ± 0.04	0.077
PDW, %	16.13 ± 0.40	16.07 ± 0.38	16.19 ± 0.40	0.115
BNP, pg/mL	92.00 (38.25–173.25)	144.00 (97.50–264.50)	42.00 (26.00–87.00)	<0.0001
ALT, U/L	22.76 ± 8.74	23.77 ± 8.61	21.67 ± 8.83	0.215
AST, U/L	20.19 ± 4.89	20.82 ± 5.12	19.50 ± 4.57	0.162
TP, g/L	72.55 ± 4.67	72.12 ± 4.40	73.02 ± 4.94	0.318
ALB, g/L	45.92 ± 2.98	45.61 ± 2.57	46.26 ± 3.36	0.258
TBIL, µmol/L	14.37 ± 7.53	16.37 ± 8.49	12.22 ± 5.66	0.004
DBIL, µmol/L	5.11 ± 2.33	5.51 ± 2.53	4.68 ± 2.03	0.065
GGT, U/L	28.00 (18.00–37.75)	28.00 (18.00–38.75)	28.00 (18.75–36.75)	0.794
CREA, µmol/L	77.70 ± 16.26	79.78 ± 18.41	75.46 ± 13.41	0.168
eGFR, mL/min/1.73 $m^{2}$	89.16 ± 16.18	85.99 ± 15.60	92.58 ± 16.24	0.034
UA, µmol/L	351.00 ± 99.88	369.38 ± 111.63	331.21 ± 79.40	0.045
GLU, mmol/L	5.94 (5.17–7.49)	5.73 (5.01–7.30)	6.17 (5.17–7.51)	0.563
GA, %	13.54 ± 3.08	13.52 ± 3.09	13.56 ± 3.09	0.950
LDH, U/L	173.44 ± 29.97	176.86 ± 31.05	169.76 ± 28.59	0.220
TG, mmol/L	1.37 (0.97–2.16)	1.36 (1.00–2.13)	1.55 (0.94–2.19)	0.780
TCHO, mmol/L	4.45 ± 1.00	4.34 ± 1.11	4.57 ± 0.86	0.227
HDL-C, mmol/L	1.19 ± 0.34	1.20 ± 0.36	1.18 ± 0.29	0.732
LDL-C, mmol/L	2.59 ± 0.80	2.48 ± 0.86	2.71 ± 0.72	0.137
LP(a), nmol/L	34.69 (14.80–69.65)	27.28 (14.80–56.22)	42.53 (15.08–70.77)	0.321
FFA, mmol/L	0.52 (0.31–0.81)	0.52 (0.38–0.73)	0.54 (0.27–0.96)	0.966
hs CRP, mg/L	0.83 (0.52–2.08)	0.89 (0.51–2.49)	0.81 (0.54–1.66)	0.638
C1q, mg/L	184.90 ± 29.56	179.79 ± 27.34	190.41 ± 31.10	0.062
HCY, µmol/L	14.19 ± 4.18	14.25 ± 3.97	14.12 ± 4.43	0.872
D-Dimer, ng/mL	59.00 (40.00–93.25)	63.00 (32.50–115.50)	57.00 (44.00–89.00)	0.574

ALB, albumin; ALT, alanine aminotransferase; AST, aspartate aminotransferase; BNP, brain natriuretic peptide; C1q, complement component 1q; TP, total protein; CREA, creatinine; DBIL, direct bilirubin; D-Dimer, D-dimer; eGFR, estimated glomerular filtration rate; FFA, free fatty acids; GA, glycated albumin; GLU, glucose; GGT, gamma-glutamyl transferase; Hb, hemoglobin; HCT, hematocrit; HCY, homocysteine; HDL-C, high-density lipoprotein cholesterol; hs CRP, high-sensitivity C-reactive protein; LDL-C, low-density lipoprotein cholesterol; LDH, lactate dehydrogenase; LDL-C, low-density lipoprotein cholesterol; LP(a), lipoprotein(a); LVZ, low voltage zone; MCH, mean corpuscular hemoglobin; MCHC, mean corpuscular hemoglobin concentration; MCV, mean corpuscular volume; MPV, mean platelet volume; PDW, platelet distribution width; PCT, plateletcrit; PLT, platelets; RDW-CV, red cell distribution width-coefficient of variation; RDW-SD, red cell distribution width-standard deviation; RBC, red blood cells; TBIL, total bilirubin; TG, triglycerides; TCHO, total cholesterol; UA, uric acid; WBC, white blood cells.

### 3.2 Univariate and Multivariate Logistic Regression Analyses of Predictors for LA LVZ

Univariate and multivariate logistic regression analyses were performed to investigate potential predictors for the presence of LA LVZ. Age, sex, type of AF, HF, LAV, LVEF, QTc, BNP, TBIL, eGFR and UA were included in the multivariate model. Multivariate logistic regression analysis revealed that persistent AF (odds ratio [OR] = 4.563, 95% confidence interval [CI]: 1.194–17.431, *p* = 0.026), LAV (OR = 1.030, 95% CI: 1.001–1.061, *p* = 0.044) and BNP (OR = 1.010, 95% CI: 1.002–1.019, *p* = 0.015) were independent predictors for the presence of LA LVZ. Detailed results for the univariate and multivariate analyses are shown in Table 3.

**Table 3.** **Univariate and multivariable logistic regression analyses of predictors for LA LVZ**.

Variable	Univariate	*p* value	Multivariate	*p* value
	OR (95% CI)		OR (95% CI)	
Age	1.039 (0.998–1.092)	0.065		
Female	1.168 (0.514–2.656)	0.710		
Persistent AF	14.930 (5.412–41.187)	<0.0001	4.563 (1.194–17.431)	0.026
HF	9.766 (1.192–90.042)	0.034		
LAV	1.051 (1.028–1.074)	<0.0001	1.030 (1.001–1.061)	0.044
LVEF	0.920 (0.860–0.983)	0.014		
QTc	1.022 (1.004–1.039)	0.016		
BNP	1.016 (1.009–1.023)	<0.0001	1.010 (1.002–1.019)	0.015
TBIL	1.102 (1.028–1.182)	0.006		
eGFR	0.974 (0.949–0.998)	0.038		
UA	1.004 (1.000–1.008)	0.048		

LA, left atrial; AF, atrial fibrillation; BNP, brain natriuretic peptide; eGFR, estimated glomerular filtration rate; HF, heart failure; LAV, left atrial volume; LVEF, left ventricular ejection fraction; QTc, corrected QT interval; TBIL, total bilirubin; UA, uric acid; OR, odds ratio; CI, confidence interval; LVZ, low-voltage zone.

### 3.3 Univariate and Multivariate Logistic Regression Analyses of Predictors for Severe LA LVZ

As shown in Table 4, LAV (OR = 1.028, 95% CI: 1.002–1.055, *p* = 0.035) and BNP (OR = 1.009, 95% CI: 1.001–1.016, *p* = 0.018) were also independent predictors of severe LA LVZ. In addition, female sex (OR = 7.161, 95% CI: 1.518–33.792, *p* = 0.013) was also found to be an independent predictor of severe LA LVZ.

**Table 4.** **Univariate and multivariate logistic regression analyses of predictors for severe LA LVZ**.

Variable	Univariate	*p* value	Multivariate	*p* value
	OR (95% CI)		OR (95% CI)	
Age	1.037 (0.993–1.082)	0.101		
Female	2.008 (0.861–4.681)	0.107	7.161 (1.518–33.792)	0.013
Persistent AF	7.508 (3.080–18.303)	<0.0001		
LAV	1.038 (1.019–1.058)	<0.0001	1.028 (1.002–1.055)	0.035
QTc	1.023 (1.004–1.043)	0.017		
BNP	1.016 (1.009–1.023)	<0.0001	1.009 (1.001–1.016)	0.018
MCH	1.550 (1.121–2.143)	0.008		
PDW	0.316 (0.108–0.928)	0.036		
TBIL	1.112 (1.039–1.192)	0.002		

LA, left atrial; AF, atrial fibrillation; BNP, brain natriuretic peptide; LAV, left atrial volume; MCH, mean corpuscular hemoglobin; PDW, platelet distribution width; QTc, corrected QT interval; TBIL, total bilirubin; OR, odds ratio; CI, confidence interval; LVZ, low-voltage zone.

### 3.4 Correlations Between Predictive Factors and LA LVZ in Males and Females

**Fig. 2.**
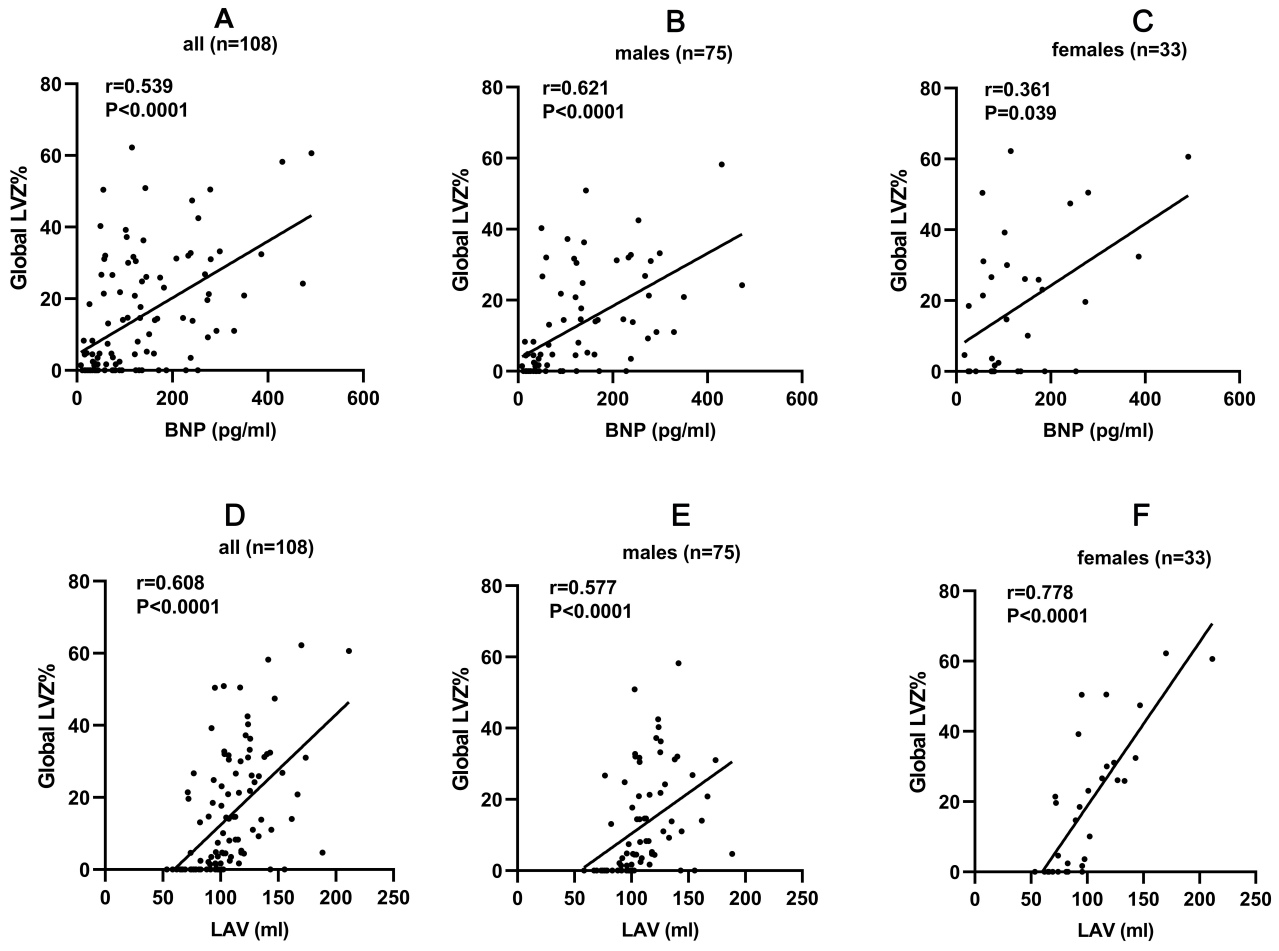
**Correlations between predictive factors and LA LVZ in male and female patients**. (A) BNP and LA LVZ in all patients; (B) BNP and LA LVZ in males; (C) BNP and LA LVZ in females; (D) LAV and LA LVZ in all patients; (E) LAV and LA LVZ in males; (F) LAV and LA LVZ in females. LA, left atrial; LVZ, low-voltage zone; LAV, left atrial volume; BNP, brain natriuretic peptide.

BNP was moderately correlated with LA LVZ in all patients (r = 0.539, *p <*0.0001), males (r = 0.621, *p <* 0.0001) and females (r = 0.361, *p* = 0.039). A strong correlation was found between LAV and LA LVZ in females (r = 0.778, *p <* 0.0001), while a moderate correlation was observed in all patients (r = 0.608, *p <* 0.0001) and in males (r = 0.577, *p <* 0.0001) (Fig. 2).

### 3.5 Regional Differences in LVZ Between Males and Females

**Fig. 3.**
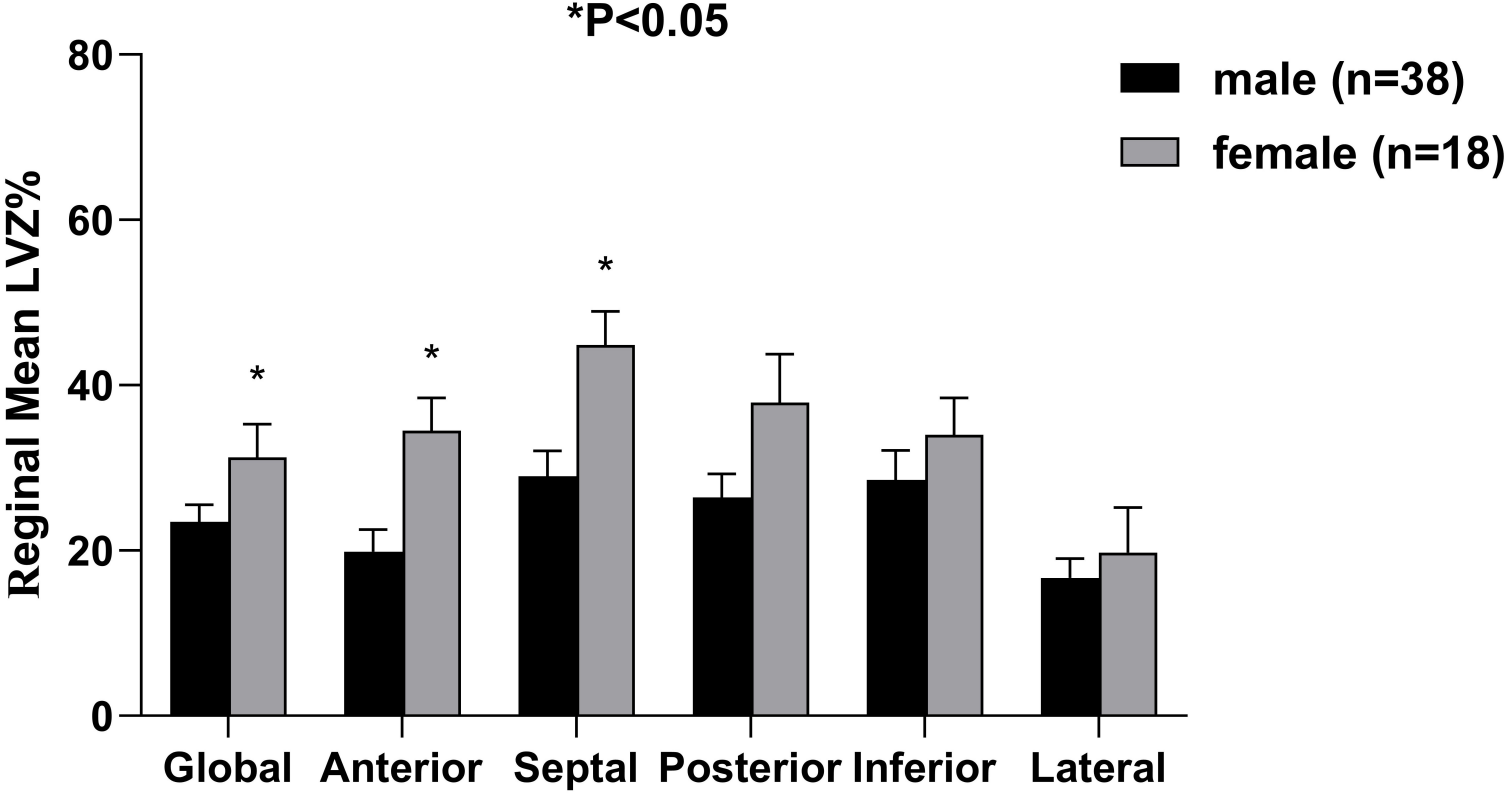
**The LA LVZ distribution in male and female patients**. The bar charts show mean and standard error values. LA, left atrial; LVZ, low-voltage zone; *, *p <* 0.05 for comparisons between males and females.

The LVZ group contained 56 patients (38 males and 18 females). The extent of global LA LVZ in females was significantly greater than that in males (32.8% ± 15.5% *vs*. 23.5% ± 12.7%, *p* = 0.021) (Fig. 3). Of note, females had larger LVZ % in the anterior wall (34.5% ± 16.7% *vs*. 20.0% ± 16.4%, *p* = 0.003) and septal wall (44.9% ± 17.1% *vs*. 29.0% ± 18.9%, *p* = 0.004). No significant sex differences were observed in the other regions.

### 3.6 Clinical Outcomes

**Fig. 4.**
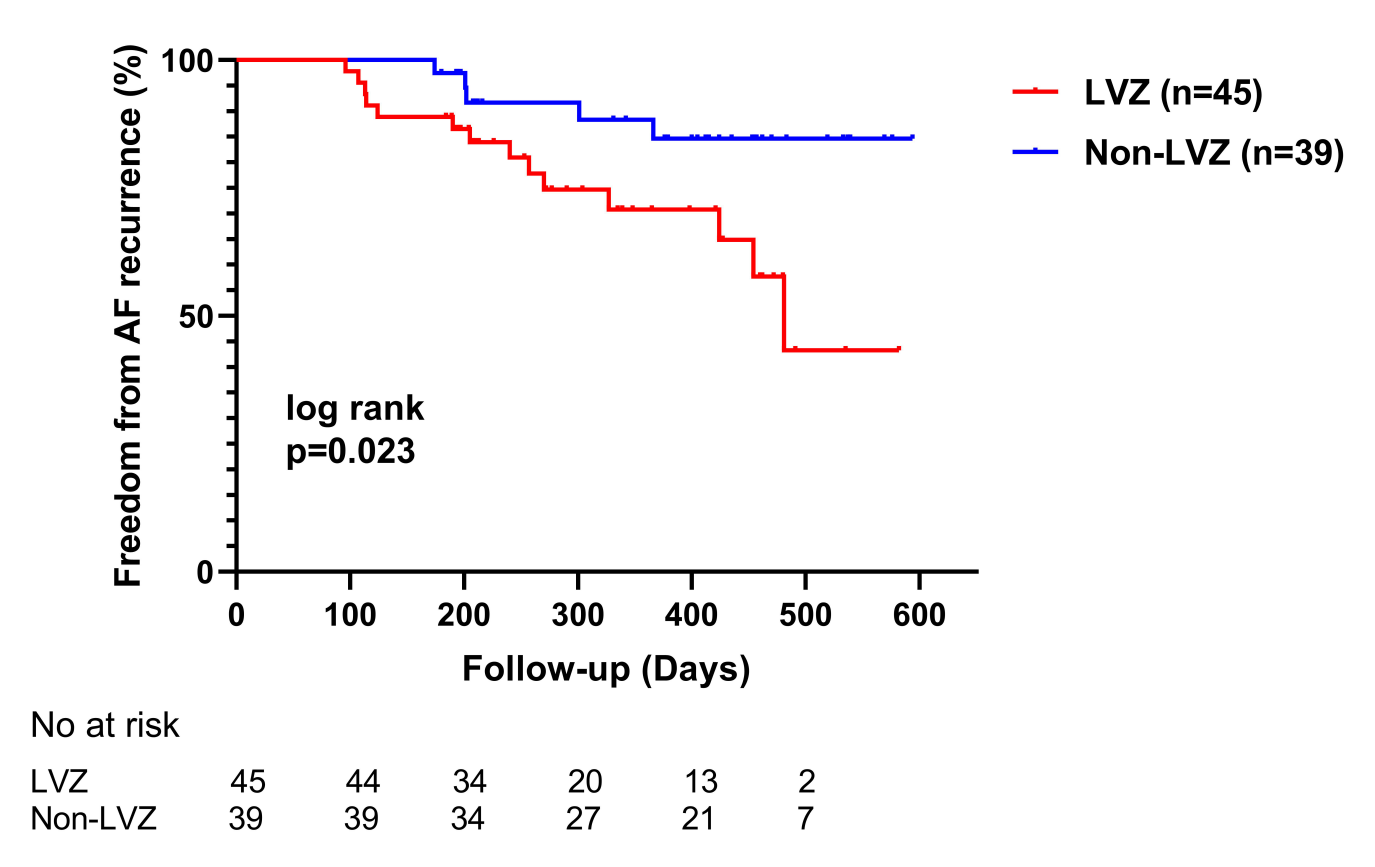
**The Kaplan-Meier curves of freedom from AF recurrence in patients with LA LVZ and those without LA LVZ**. AF, atrial fibrillation; LA, left atrial; LVZ, low-voltage zone.

For the investigation of AF recurrence, 24 patients with <6 months follow-up were excluded, leaving 84 patients for analysis. The median follow-up time was 339 days, and 19 patients (22.6%) suffered AF recurrence after RFCA. Kaplan-Meier analysis that presented in Fig. 4 showed that AF recurrence was significantly higher in patients with LA LVZ than in those without LA LVZ (31.3% for LVZ *vs*. 12.8% for non-LVZ, *p* = 0.023). Furthermore, as shown in Table 5, after adjusting for age, sex, type of AF, LAV, and BNP, the extent of LA LVZ was still a risk factor for AF recurrence (hazard ratio [HR]: 1.945; 95% CI: 1.003–1.088; *p* = 0.034). The baseline characteristics of the 84 patients were shown in **Supplementary Table 1**.

**Table 5.** **Univariate and multivariate Cox regression analyses of risk factors for AF recurrence**.

Variable	Univariate	*p* value	Multivariate	*p* value
	HR (95% CI)		HR (95% CI)	
Age	1.094 (1.038–1.153)	0.001	1.118 (1.042–1.200)	0.002
Female	1.658 (0.651–4.218)	0.289		
Persistent AF	3.009 (1.183–7.656)	0.021		
LAV	1.019 (1.007–1.032)	0.002		
BNP	1.006 (1.003–1.010)	<0.0001		
Extent of LA LVZ	1.049 (1.024–1.074)	<0.0001	1.045 (1.003–1.088)	0.034

AF, atrial fibrillation; BNP, brain natriuretic peptide; LA, left atrial; LAV, left atrial volume; LVZ, low-voltage zone; HR, hazard ratio; CI, confidence interval.

## 4. Discussion

The results of this study showed that persistent AF, LAV and BNP are independent predictors for the presence of LA LVZ, and that female sex, LAV and BNP are independent predictors of severe LA LVZ. Furthermore, females had a significantly higher incidence of LVZ, particularly in the anterior and septal walls. We also confirmed that patients with LA LVZ had a higher risk of recurrent AF.

Pulmonary vein isolation is the first-line treatment for patients with symptomatic and drug-refractory AF. However, AF recurrence occurs in 22% to 50% of patients, with up to 70% showing persistent AF during follow-up [19, 20, 21]. A possible explanation for these observations could be the presence of non-pulmonary vein (PV) foci caused by LA fibrosis. Currently, only a few methods are available to evaluate LA fibrosis. Several studies have shown that late gadolinium enhancement-magnetic resonance imaging is the gold standard for evaluating LA fibrosis [22, 23]. However, this method is costly, cumbersome, and can only be carried out in experienced imaging centers, thus limiting its practical value in the clinic. High-density electro-anatomic mapping is a viable alternative that can be used to assess the extent of LA fibrosis by mapping the LA LVZ. Previous studies have shown that the presence of LA LVZ is closely associated with AF recurrence and increased stroke incidence in patients with AF [24, 25, 26]. Ablation that targets the LA LVZ can improve the long-term prognosis of patients [27]. The identification of practical predictors for the presence of LA LVZ is crucial because it would allow more accurate evaluation of prognosis and help in the selection of a suitable ablation strategy. However, since the evaluation of LA LVZ requires electrophysiological analysis with intracardiac electrodes, it is important to find alternative methods to identify LA LVZ.

In the present study, persistent AF was found to be an independent predictor of LA LVZ. This is consistent with previous reports that the LA LVZ area in patients with persistent AF is larger than in patients with paroxysmal AF [18]. This may explain the high recurrence rate after PV isolation (PVI) alone in patients with persistent AF, and suggests that ablation targeted at the LA LVZ could improve their long-term outcome [28, 29].

Previous studies have identified BNP as a predictor of new onset AF and of AF recurrence following catheter ablation [30, 31, 32]. BNP can also predict the occurrence of stroke and other complications in patients with AF [33, 34]. However, so far there have been few studies on the relationship between BNP levels and atrial fibrosis [35]. Moreover, possible correlations between the severity of LA LVZ and BNP levels according to sex have yet to be reported. BNP is a common index that reflects cardiac load in clinical practice. Increased LA pressure and stretch during episodes of AF can promote atrial secretion of BNP, independently of ventricular secretion [36, 37]. Some authors have proposed that atrial stretch is an important stimulus for fibrosis [38], suggesting the BNP level can in theory reflect the degree of LA fibrosis. In the present cohort, BNP was a strong predictor of the presence of LA LVZ, as well as of severe LA LVZ. The BNP level was also moderately correlated with LA LVZ in the overall patient cohort. Our results further demonstrate that the BNP level is associated with the degree of atrial fibrosis.

New biomarkers have become a very active area of research in recent years. An increasing number of biomarkers have been reported to be closely correlated with LA LVZ, including *miRNA-21*, soluble suppression of tumorigenicity 2 protein (ST2), interleukin-17A, and interferon-γ [39, 40, 41]. Although promising, these biomarkers have not been widely used in clinical practice, and BNP remains by far the most established and clinically accessible parameter.

In the present study we demonstrated that LAV is an independent predictor of both LA LVZ and severe LA LVZ, consistent with the results from other groups [42, 43]. We also showed that LAV was moderately correlated with LA LVZ in the overall patient cohort and in males, and strongly correlated with LA LVZ in females. To our knowledge, this sex difference has not been reported in previous studies.

In addition to focusing on particular independent predictors, recent studies have also developed models to predict the presence of LA LVZ. In 2015, Kosiuk *et al*. [44], created and validated the DR-FLASH score based on diabetes mellitus, renal dysfunction, persistent AF, LA diameter >45 mm, age >65 years, female sex, and hypertension. These authors concluded that a DR-FLASH score >3 was strongly correlated with the presence of LA LVZ. Following this, the acute APPLE score, ANP score and modified APPLE score were subsequently proposed [45, 46, 47]. Although each score is based on different factors, they showed no significant difference in their ability to predict LA LVZ. The use of these scores can help to identify individuals that require PVI alone or additional, targeted LVZ ablation. They can also assist with the selection of an appropriate ablation strategy prior to RFCA. However, the occurrence and development of LA LVZ is probably multifactorial and therefore a universal scoring system that is predictive of LA LVZ will be difficult to develop.

The incidence, clinical manifestations, and prognosis of AF differ between males and females. It is well established that females have a lower incidence of AF than males, but are more likely to experience AF recurrence after catheter ablation, as well as stroke, heart failure, and even death [48, 49, 50]. However, the mechanisms that underlie sex differences in AF have not been fully elucidated. Some studies have shown that female sex is predictive of LA LVZ [43]. However, sex differences in the distribution of LA LVZ have not been reported in previous studies. In the current study, female sex was an independent predictor of severe LA LVZ. Females were also found to have a significantly greater extent of LA LVZ than males, especially in the anterior and septal walls, suggesting they may have more severe LA fibrosis than males. This observation may explain why female patients are more likely to experience AF recurrence, stroke and other complications than male patients, and could also be used to inform the choice of ablation strategy.

Finally, we explored the relationship between LA LVZ and AF recurrence. Patients with LA LVZ were found to have a higher risk of recurrent AF and the extent of LA LVZ was a powerful risk factor for AF recurrence, in line with previous studies [26, 40]. The finding strongly suggested that it is highly desirable to explore independent predictor of LA LVZ. However, additional large-scale studies are needed to confirm this finding.

### Limitations

This was a single-center study with a relatively small sample size, and hence further validation is needed to determine whether these findings can be generalized. Secondly, left atrial appendage (LAA) was not adequately modeled in a significant number of cases, which could affect the accuracy of the results. Thirdly, some of the cases in this study were mapped under AF rhythm. Although previous studies have confirmed the feasibility of using 0.3 mV as the threshold for LVZ under AF rhythm, the effect of this method on the LVZ area is unknown.

## 5. Conclusions

In conclusion, this study demonstrated that persistent AF, LAV and BNP were associated with LA LVZ in patients with AF. Furthermore, we found that female sex, LAV and BNP were associated with severe LA LVZ. Female patients exhibited greater extent of LVZ than male patients, especially in the anterior and septal walls. Finally, the presence of LA LVZ was strongly associated with an increased risk of AF recurrence and the extent of LA LVZ had an independent predictive value for AF recurrence after RFCA.

## Data Availability

The datasets used and analyzed during the current study are available from the corresponding author on reasonable request.

## Supplementary Material

Supplementary material associated with this article can be found, in the online version, at https://doi.org/10.31083/j.rcm2411324.
