# Supplementary material for: Predictors of Low Voltage Zone and Sex Differences in Low Voltage Zone Distribution in Patients with Atrial Fibrillation
Source: Rev Cardiovasc Med. 2023 Nov 23;24(11):324. doi: 10.31083/j.rcm2411324 (PMC11272876; doi:10.31083/j.rcm2411324)
Supplement: Supplementary file 1 [file 2153-8174-24-11-324-s1.docx]

Supplementary Table 1. Baseline characteristics of patients included in follow-up

| **Characteristic** | **Total**  **(n=84)** | | **Recurrence**  **(n=19)** | **No recurrence**  **(n=65)** | ***P* value** |
| --- | --- | --- | --- | --- | --- |
| Age, years | 58.48±10.17 | | 64.68±8.80 | 56.66±9.87 | **0.002** |
| Female, n (%) | 24 (28.6) | | 7 (36.8) | 17 (26.2) | 0.364 |
| BMI, kg/m2 | 25.93±2.90 | | 26.12±2.80 | 25.87±2.94 | 0.949 |
| Persistent AF, n (%) | 33 (39.3) | | 12 (63.2) | 21 (32.3) | **0.015** |
| HTN, n (%) | 46 (54.8) | | 11 (57.9) | 35 (53.8) | 0.755 |
| DM, n (%) | 29 (34.5) | | 9 (47.4) | 20 (30.8) | 0.181 |
| CAD, n (%) | 13 (11.9) | | 3 (15.8) | 7 (10.8) | 0.848 |
| HF, n (%) | 8 (9.5) | | 4 (21.1) | 4 (6.2) | 0.133 |
| Stroke, n (%) | 13 (15.5) | | 5 (26.3) | 8 (12.3) | 0.138 |
| Smoking, n (%) | 18 (21.4) | | 4 (21.1) | 14 (21.5) | 0.964 |
| Drinking, n (%) | 15 (17.9) | | 2 (2.4) | 13 (15.5) | 0.543 |
| LAD, mm | 39.79±5.21 | | 43.00±4.91 | 38.85±4.94 | **0.002** |
| LVEF, % | 62.14±7.23 | | 60.74±7.74 | 62.55±7.08 | 0.338 |
| LAV, ml | 107.58±29.38 | | 127.90±35.16 | 101.64±24.76 | **0.003** |
| BNP, pg/ml | 99.00 (37.25-182.25) | | 233.00 (107.00-299.00) | 77.00 (36.00-142.50) | **0.003** |
| eGFR, ml/min/1.73m^2^ | | 88.81±16.81 | 82.37±16.91 | 90.70±16.43 | 0.057 |
| LA LVZ, % | 8.30 (0-26.45) | | 26.80 (4.41-33.21) | 4.71 (0-18.11) | **0.007** |

AF: atrial fibrillation; BMI: body mass index; BNP: brain natriuretic peptide; CAD: coronary artery disease; DM: diabetes mellitus; eGFR: estimated glomerular filtration rate; HTN: hypertension; HF: heart failure; LAD: left atrial diameter; LAV: left atrial volume; LVZ: low voltage zone; LVEF: left ventricular ejection fraction.
